# AhR and HIF-1*α* Signaling Pathways in Benign Meningioma under Hypoxia

**DOI:** 10.1155/2023/6840271

**Published:** 2023-06-03

**Authors:** Maria L. Perepechaeva, Lyubov S. Klyushova, Alevtina Y. Grishanova

**Affiliations:** Institute of Molecular Biology and Biophysics, Federal Research Center of Fundamental and Translational Medicine, Timakova Str. 2, Novosibirsk 630117, Russia

## Abstract

The role of hypoxia in benign meningiomas is less clear than that in the malignant meningiomas. Hypoxia-induced transcription factor 1 subunit alpha (HIF-1*α*) and its downstream signaling pathways play a central role in the mechanism of hypoxia. HIF-1*α* forms a complex with the aryl hydrocarbon receptor nuclear translocator (ARNT) protein and can compete for ARNT with aryl hydrocarbon receptor (AhR). In this work, the status of HIF-1*α*- and AhR-dependent signaling pathways was investigated in World Health Organization (WHO) grade 1 meningioma and patient-derived tumor primary cell culture under hypoxic conditions. mRNA levels of *HIF-1α*, *AhR*, and of their target genes as well as of *ARNT* and nuclear receptor coactivator *NCOA2* were determined in tumor tissues from patients in whom the tumor was promptly removed either with or without prior endovascular embolization. Using the patient-derived nonembolized tumor primary cell culture, the effects of a hypoxia mimetic cobalt chloride (CoCl_2_) and an activator of the AhR signaling pathway benzo(*α*)pyrene (B[a]P) on mRNA levels of *HIF-1α*, *AhR*, and their target genes were investigated. Our findings show active functioning of AhR signaling in meningioma tissue of patients with tumor embolization and crosstalk between HIF-1*α* and AhR signaling in meningeal cells under hypoxia.

## 1. Introduction

Meningiomas are the most common tumors of the central nervous system and arise from arachnoid cells of the meninges. These tumors constitute at least a third of all intracranial tumors and half of primary benign tumors [1, 2]. WHO classifies meningiomas into benign grade (WHO grade 1, WHOGr1), atypical grade (WHOGr2), and malignant grade (WHOGr3), which represent 80.3%, 17.9%, and 1.6% of all meningioma cases, respectively [2]. According to other data, up to 85–90% of meningiomas are benign [3]. Since 2021, the WHO classification of meningiomas takes into account such molecular biomarkers as NF2, AKT1, TRAF7, SMO, PIK3CA, KLF4, SMARCE1, BAP1, H3K27me3, TERT, CDKN2A, and CDKN2B [4]. Nonetheless, meningiomas are still subtyped primarily by means of histological criteria [5].

Hypoxia is insufficient oxygenation, which is common for solid tumors [6–8]. There are few studies aimed at elucidating the role of hypoxia in the biology of meningiomas [9–12], and this kind of research usually deals with clarification of the involvement of hypoxia-regulated molecules in the phenotype of malignant meningiomas [9–12]. The role of hypoxia in the biology of benign meningiomas is less clear, although these tumors are often life-threatening and show variable behavior and aggressiveness with an elevated recurrence rate [2, 3, 13].

In the mechanism of hypoxia at the molecular level, the central role is played by proteins HIF-1, HIF-2, and HIF-3 (hypoxia-induced factors) and by the signaling pathways regulated by them, which are related to development, metabolism, cell proliferation, inflammation, and processes mediating angiogenesis and oncogenesis [14, 15]. HIF-1*α* is an oxygen-regulated member of the bHLH/PAS family of transcription factors and governs the expression of genes whose products participate in homeostasis during changes in oxygen concentration; besides, this protein mediates cellular responses to hypoxia by forming a heterodimer with ARNT in the nucleus, and this dimer launches the transcription of target genes [16].

Aside from the HIF-1-dependent signaling cascade, other pathways can be activated by hypoxic conditions. AhR, which—just as HIF-1*α*—belongs to the bHLH/PAS family of transcription factors, is important both for normal cell physiological processes and for tumor progression because AhR partakes in the molecular cascades that inhibit cell proliferation, differentiation, and apoptosis [17–19]. In particular, AhR can regulate angiogenesis during tumor progression [20].

An increasing number of studies show that there are overlaps between the HIF-1*α* and AhR pathways in terms of the control over biological functions, including tumor progression [21–23]. It is known that the activation and inhibition of bHLH-PAS family proteins (which include HIF-1*α*, ARNT, and AhR) are governed by transcriptional coactivator NcoA2 through protein–protein interactions [24]. AhR, just as HIF-1*α*, forms a DNA-binding heterodimeric complex with the ARNT protein to regulate the expression of target genes [25], and AhR competes with HIF-1*α* for ARNT; this mechanism may be responsible for the antagonistic relation between the AhR and HIF-1*α* signaling pathways [24, 26]. Hypoxia can weaken AhR ligand-driven activation of the AhR signal transduction pathway, whereas treatment with AhR ligands can attenuate HIF-1*α*-mediated responses to hypoxia [21]. Competition between AhR and HIF-1*α* during AhR inhibition can promote angiogenesis via HIF-1*α* signal transduction [23].

The biological understanding of the response to hypoxia and of its effects on various signaling pathways is currently inadequate. Hypoxia determines many parameters of malignant progression of tumors and contributes to this progression [7], but hypoxia-activated signaling cascades may also participate in the suppression of tumor growth [27, 28].

In more than half of the cases, active HIF-1 is detectable in benign meningiomas, and hypoxia—and hence HIF-1 upregulation—is associated with the recurrence of meningiomas and their progression [9].

Endovascular embolization, in other words, selective obliteration of tumor vasculature with preservation of the blood supply to healthy tissues, is a standard procedure in the treatment of meningiomas [29, 30] because such devascularization of a tumor beforehand facilitates the surgical operation and reduces blood loss [31, 32]. Nevertheless, there is controversy regarding the indications and clinical benefits of this procedure [29, 30, 32]. Endovascular embolization evidently causes ischemia and promotes tumor tissue hypoxia, but it is not known how artificially induced hypoxia affects changes in meningioma cells at the molecular level. It is necessary to identify the factors able to direct hypoxia-driven changes of the functioning of molecular cascades either toward a prooncogenic pathway or toward a proapoptotic pathway that suppresses the tumor.

In this study, we focus on AhR as a potential participant in the regulation of the malignant progression of meningeal cells under hypoxic conditions. The purposes of the study were as follows: (1) to test whether preoperative embolization of WHOGr1 meningiomas affects the AhR signaling pathway and (2) to investigate the impact of HIF-1*α* and AhR inducers on HIF-1*α* and AhR signaling pathways and determine whether there is crosstalk between these pathways in meningioma cells.

Here, we report the results of a study on the expression of key genes of the HIF-1*α* and AhR signaling pathways, of their heterodimerization partner *ARNT*, of nuclear receptor coactivator *NcoA2*, and of HIF-1*α* target gene vascular endothelial cell growth factor *VEGF-A*, proto-oncogene *c-Myc*, and glucose transporter *GLUT1* in the tissue of benign (WHOGr1) meningiomas. Also presented are the findings about the impact of a hypoxia mimetic (CoCl_2_) and of an activator of the AhR signaling pathway (B[a]P) on mRNA levels of *HIF-1α*, *AhR*, and of their target genes in primary culture of patient-derived nonembolized tumor cells.

## 2. Materials and Methods

### 2.1. Sample Collection

For this study, we used surgically resected human meningioma biopsy tissue samples from 18 patients with benign (WHOGr1) meningioma who underwent surgical treatment in the Neurosurgical Department of the Neurosurgical Center at a private clinic (Clinical Hospital Novosibirsk station RZD-Medicine). The patients' age was 55 ± 9 years (mean ± SD), and the male-to-female ratio was 28%/72%. In all patients, the tumor was a primary lesion. Histopathological diagnosis revealed that there were three histological subtypes of meningioma: meningotheliomatous (eight tumors), fibroblastic (four tumors), and transitional (mixed) (six tumors).

There were two groups of patients with meningioma: patients who had the tumor removed without prior endovascular embolization (*n* = 8) and patients who had the tumor removed after endovascular embolization (*n* = 10).

The tumor samples were collected during surgical tumor resection. The biopsy tissue samples were snap-frozen in liquid nitrogen immediately after the surgical operation and stored at −80°C until mRNA analysis or were placed into a microtube containing oxygenated cold phosphate-buffered saline (PBS was purged with oxygen before transportation) and were transported to the laboratory no later than 2 h afterwards for the preparation of primary cell cultures.

### 2.2. Ethical Approval

All procedures involving human participants were performed in accordance with the ethical standards of the 1964 Helsinki Declaration and its later amendments or comparable ethical standards. The study protocol was approved by the Committee for Biomedical Ethics of the Institute of Molecular Biology and Biophysics of the Federal Research Center of Fundamental and Translational Medicine (IMBB FRC FTM) and was certified not being a risk to the patients' health. Informed consent was obtained from all participants of the study.

### 2.3. Preparation of Primary Cell Cultures

The tissue was washed with PBS and crushed, and a 0.1% solution of type I collagenase was added and incubated for 30 min at 37°C. The resulting cell suspension was filtered to remove large pieces of tissue and pelleted by centrifugation at 1000 rpm for 5 min. The precipitate was resuspended in the RPMI 1640 medium (supplemented with 10% or less of fetal calf serum, 100 U/mL penicillin, and 100 U/mL streptomycin, hereafter complete RPMI 1640 medium) and placed in culture flasks.

The cells were seeded at a high concentration of calf fetal serum (10%), and after cell attachment to the plastic substrate (culture flask), serum concentration was reduced to 3%. It was necessary to vary the serum concentration because at its high concentration (10%), the growth of fibroblasts can accelerate [33, 34].

The cells were cultured in a CO_2_ incubator (37°C, 5% CO_2_, and 80% humidity), and the growth medium was refreshed every 3 days. Only when the monolayer of the cells reached 80% confluence were the cells detached using a 0.25% trypsin solution and subcultured in a 1 : 3 ratio. The third passage of the cultured cells was used for the experiments.

All cells were checked periodically for mycoplasma contamination using the MycoSensor qPCR Assay Kit (Agilent, USA) according to the manufacturer's instructions.

### 2.4. Cell Viability Assay

Cell viability was evaluated by Hoechst/propidium iodide staining by a standard method as previously described [35]. For the identification of live, apoptotic, and dead cells, treated cells and control cells were stained with a mixture of fluorescent dyes Hoechst 33342 (Sigma-Aldrich) and propidium iodide (Invitrogen) for 30 min at 37°C. IN Cell Analyzer 2200 (GE Healthcare, UK) was employed to analyze at least six visual fields per well at 200x magnification with automatic imaging in bright-field mode and in fluorescence channels. IN Cell Investigator image analysis software was utilized to identify live, apoptotic, and dead cells. All data shown are the mean of three wells. The quantitative data were expressed as the mean ± SE. The experiments were performed in triplicate.

### 2.5. Cell Phenotyping Assay

The epithelial type of the resulting cultured cells was confirmed by an immunofluorescent assay of osteopontin (OPN) expression [36]. The groups were as follows: a control, 200 *μ*M CoCl_2_ added to the incubation medium, and 200 *μ*M CoCl_2_+1 *μ*M B[a]P added to the incubation medium.

For immunocytochemical analysis, the culture medium was discarded, and the cells were rinsed with PBS and fixed with 4% paraformaldehyde for 10 min at room temperature. Permeabilization was carried out by means of a Triton X-100 solution, and 1% BSA served as a blocker. Then, the cells were incubated with an anti-OPN rabbit polyclonal antibody (Abcam cat. # *ab844*) (primary antibody, dilution 1 : 200) for 1 h at room temperature. After that, a secondary fluorescently labeled antibody (rabbit anti-human IgG (H+L) cross-adsorbed secondary antibody, DyLight 550, Invitrogen, 1 : 1000) was used. The incubation lasted for 30 min at room temperature. Phase contrast and fluorescent images of cells were assessed on IN Cell Analyzer 2200 (GE Healthcare, UK) in bright-field mode and in DAPI and Cy3 channels. The experiments were performed in triplicate.

### 2.6. Treatment of Primary Culture with Hypoxia Mimetic CoCl_2_

To simulate hypoxic conditions, CoCl_2_ was used, the mechanism of action of which is based on an increase in the intracellular concentration of HIF-1*α* [37]. The primary cells were cultured in 96-well plates in the complete RPMI 1640 medium in the CO_2_ incubator. After 1 day, CoCl_2_ was added at a concentration of 125, 250, 500, or 1000 *μ*M and incubated for 24 h.

At least 2000 cells were evaluated at each data point. The percentage of cells was estimated relative to the number of cells in the control. The percentages of live, apoptotic, and dead cells were calculated from the total number of cells at each data point.

The mRNA expression of genes *HIF-1α*, *VEGF-A*, *GLUT1*, *AhR*, *ARNT*, and *CYP1B1* was determined in cells exposed to 200 *μ*M CoCl_2_.

### 2.7. Treatment of Primary Cultured Cells with AhR Activator B[a]P

The primary cells were cultured in 96-well plates in the complete RPMI 1640 medium in the CO_2_ incubator. A day later, 1 *μ*M B[a]P (reagent grade pure, *Fluka* AG, *Buchs SG*, Switzerland, cat. # bz 394701) was added, and after incubation for 24 h, the mRNA expression of genes *HIF-1α*, *VEGF-A*, *GLUT1*, *AhR*, *ARNT*, and *CYP1B1* was determined in the cells. We chose the concentration of B[a]P at which there was an obvious effect on the studied genes without severe cytotoxicity.

### 2.8. Cotreatment of the Primary Cultured Cells with CoCl_2_ and B[a]P

The primary cells were cultured in 96-well plates in the complete RPMI 1640 medium in the CO_2_ incubator. A day later, 200 *μ*M CoCl_2_ and 1 *μ*M B[a]P were added, and after incubation for 24 h, the mRNA expression of genes *HIF-1α*, *VEGF-A*, *GLUT1*, *AhR*, *ARNT*, and *CYP1B1* was determined in the cells.

### 2.9. Isolation of RNA

Total RNA from meningioma tissue was isolated with the RNeasy Plus Micro Kit (Qiagen) according to the manufacturer's instructions. The isolation of RNA from the meningioma primary cultured cells was carried out with the TRI-Reagent Kit (Ambion) according to the manufacturer's instructions. The RNA was treated with DNase (Promega, USA) and precipitated. The RNA pellets were dissolved in 1 mM sodium citrate buffer (pH 6.5) containing 1x RNASecure Reagent. The RNA samples were stored at –20°C until use.

### 2.10. Reverse Transcription and Polymerase Chain Reaction (PCR) Analysis of the Meningioma Tissue Samples and Cultured Cells

The RNA was reverse-transcribed into complementary DNA by a reverse transcription reaction with M-MuLV reverse transcriptase (Promega, USA) according to the manufacturer's instructions. mRNA levels of genes *HIF-1α*, *AhR*, *ARNT*, *VEGF-A*, *GLUT1*, *NcoA2*, and *CYP1B1* were evaluated by real-time PCR according to the TaqMan principle on an iCycler CFX96 real-time PCR machine (Bio-Rad Laboratories). *EIF2B1* served as a housekeeping reference gene [38]. The primers were designed in the Example 3 Plus software. The sequences of primers are *HIF-1α* (forward) 5′-CCCAATGGATGATGACTTCC-3′, (reverse) 5′-TGGGTAGGAGATGGAGATGC-3′, and (probe) 5′(FAM)-GAAAGCGCAAGTCCTCAAAG-(BHQ1)3′; *AhR* (forward) 5′-CGGAGGCCAGGATAACTGTA-3′, (reverse) 5′-GAAATTCAGCTCGGTCTTCG-3′, and (probe) 5′(FAM)-CAGGCTCTGAATGGCTTTGT-(BHQ1)3′; *ARNT* (forward) 5′-AACCTCACTTCGTGGTGGTC-3′, (reverse) 5′-CAATGTTGTGTCGGGAGATG-3′, and (probe) 5′(FAM)-CTAGTGGCCATTGGCAGATT-(BHQ1)3′; *VEGF-A* (forward) 5′-CCCACTGAGGAGTCCAACAT-3′, (reverse) 5′-TTTCTTGCGCTTTCGTTTTT-3′, and (probe) 5′(FAM)-AGGCCAGCACATAGGAGAGA-(BHQ1)3′; *GLUT1* (forward) 5′-GCGGAATTCAATGCTGATGAT-3′, (reverse) 5′-CAGTTTCGAGAAGCCCATGAG-3′, and (probe) 5′(FAM)-CTGGCCTTCGTGTCCGCCGT-(BHQ1)3′; *c-Myc* (forward) 5′-CCTCCACTCGGAAGGACTATC-3′, (reverse) 5′-AAGCTCCGTTTTAGCTCGTTC-3′, and (probe) 5′(FAM)-AACAACCGAAAATGCACCA-(BHQ1)3′; *NcoA2* (forward) 5′-AATGCATCAGCAACAGCAAG-3′, (reverse) 5′-ATAAGTGGGCTCTGGGGAGT-3′, and (probe) 5′(FAM)-TGCCAGCAACTATGAGCAAC-(BHQ1)3′; *CYP1B1* (forward) 5′-TGATGGACGCCTTTATCCTC-3′, (reverse) 5′-CCACGACCTGATCCAATTCT-3′, and (probe) 5′(FAM)-GGCTGGATTTGGAGAACGTA-(BHQ1)3′; and *EIF2B1* (forward) 5′-GGTGCTAGATGCTGCTGTCG-3′, (reverse) 5′-ATCTGGGACGTCTTGCTGGT-3′, and (probe) 5′(FAM)-TGCCAAAGCACAGAACAAAC-(BHQ1)3′.

The reaction mixture consisted of either qPCRmix-HS buffer (Evrogen, Russia) or BioMaster HS-qPCR buffer (Biolabmix, Russia), a primer mix consisting of 100 nM probe (final concentration), 400 nM forward and reverse primers each (final concentration), and 500 ng of cDNA.

The reaction was conducted under the following conditions: heating at 95°C for 3 min, then 40 cycles of denaturation at 95°C for 15 s, and annealing/extension at 60°C for 30 s. All samples were analyzed in triplicate in two independent experiments. Separate standard curves were built for each experiment by means of serial dilutions (1 : 3 to 1 : 27) of a cDNA standard (see below). For each sample, the amount of cDNA of a target gene was normalized to that of the reference gene.

To analyze the data, the calibration graph method was employed in the software supplied with the thermal cycler. Aliquots from all cDNA samples were mixed, and this “average” solution (cDNA standard) was used to construct the calibration curves for measurement of relative cDNA levels of the genes of interest and of the reference gene.

### 2.11. Western Blot Analysis

This immunoblot assay involved antibodies against HIF-1*α* and AhR (with normalization to *β*-actin). The lysate proteins from the tumor samples were separated by electrophoresis in an SDS 8% polyacrylamide gel and transferred to a polyvinylidene difluoride membrane via semidry transfer in a Trans-Blot Turbo system (Bio-Rad). Antibodies against HIF-1*α* (cat. # ab16066, Abcam) and against AhR (ab2770, Abcam) served as primary antibodies. Peroxidase-labeled secondary antibodies were utilized too. The results were visualized by chemiluminescence using the ECL Kit (Bio-Rad Laboratories). The experiments were performed in triplicate.

### 2.12. Statistical Analysis

This procedure was carried out in the Statistica software (StatSoft Inc.). To evaluate the significance of differences between groups of the samples, one-way analysis of variance (ANOVA) was performed, and the Bonferroni correction served as a post hoc test.

## 3. Results

### 3.1. Expression of *HIF-1α*, of HIF-*α* Target Genes (*VEGF-A*, *GLUT1*, and *c-Myc*), and of *AhR*, *ARNT*, and *NcoA2* in Tissue Samples of Benign Meningiomas

**Figure 1:**
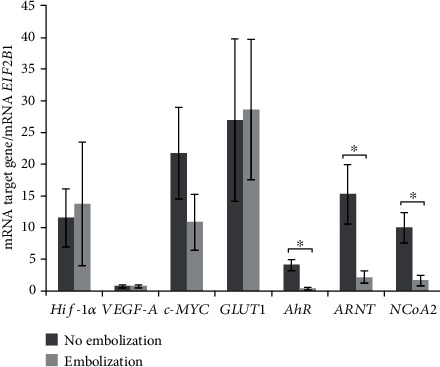
mRNA levels of *HIF-1α*, *AhR*, *ARNT*, *VEGF-A*, *c-Myc*, *GLUT1*, and *NcoA2* in nonembolized and endovascularly embolized human meningiomas. The data are presented as the mean ± SE; ^∗^*p* < 0.05. For nonembolized meningiomas: *n* = 8 for *HIF-1α*, *AhR*, *ARNT*, and *VEGF-A* and *n* = 7 for *c-Myc* and *GLUT1*. For embolized meningiomas: *n* = 10 for *AhR* and *VEGF-A* and *n* = 9 for *HIF-1α*, *ARNT*, *c-Myc*, and *GLUT1*. One-way analysis of variance (ANOVA) was performed, and the Bonferroni correction served as a post hoc test.

The analysis of the expression of *HIF-1α* and HIF-*α* target genes (*VEGF-A*, *GLUT1*, and *c-Myc*) in meningioma tissue samples obtained during surgical removal of the tumor in patients either with or without prior endovascular embolization revealed no statistically significant differences in the mRNA levels of these genes between the nonembolized and embolized meningiomas (Figure 1).

At the same time, mRNA levels of *AhR*, *ARNT*, and *NcoA2* in the meningiomas subjected to hypoxia as a result of the embolization were statistically significantly lower than those in the nonembolized meningiomas (Figure 1).

**Figure 2:**
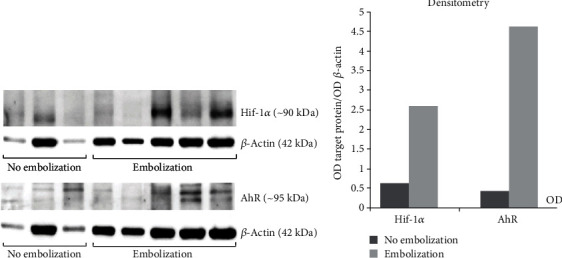
Western blot analysis of HIF-1*α* and AhR protein levels in nonembolized and endovascularly embolized human meningiomas. This immunoblot assay involved antibodies against HIF-1*α* and AhR with normalization to *β*-actin. One-way analysis of variance (ANOVA) was performed, and the Bonferroni correction served as a post hoc test. For nonembolized meningiomas: *n* = 3; for embolized meningiomas: *n* = 5. The data are presented as the mean ± SE.

In the analysis of HIF-1*α* and AhR expression at the protein level, there were no statistically significant differences in the expression of these proteins between nonembolized and embolized meningiomas (Figure 2).

### 3.2. Expression of *HIF-1α*, HIF-1*α* Target Genes (*VEGF-A* and *GLUT1*), and *ARNT*, *AhR*, and *CYP1B1* in Patient-Derived Tumor Primary Cell Lines

#### 3.2.1. Cell Viability and Phenotyping Assays

**Figure 3:**
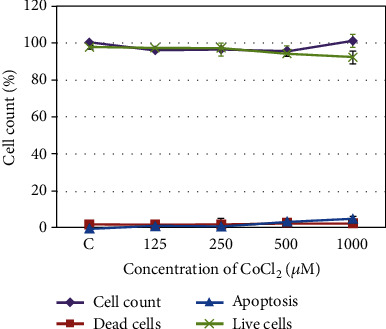
Assessment of CoCl_2_ toxicity to meningioma primary cultured cells. The percentages of live (L), apoptotic (AP), and dead cells (D) are presented for the cells incubated for 24 h with various concentrations of CoCl_2_. The number of cells incubated without CoCl_2_ (С, control) was set to 100%. The curves indicate changes in numbers of cells (live, dead, or apoptotic at each concentration of CoCl_2_ relative to the control). The data are presented as the mean ± SE.

Primary cell cultures were derived from six benign WHOGr1 nonembolized meningiomas of the meningotheliomatous histological subtype. CoCl_2_ was used to simulate hypoxic conditions. We found a nontoxic concentration of CoCl_2_ after treatment of the cell lines with various doses. The experiment showed that in the range of CoCl_2_ concentrations from 125 to 250 *μ*M, the percentage of live cells in the culture did not change, and apoptotic and dead cells were not detectable (Figure 3).

**Figure 4:**
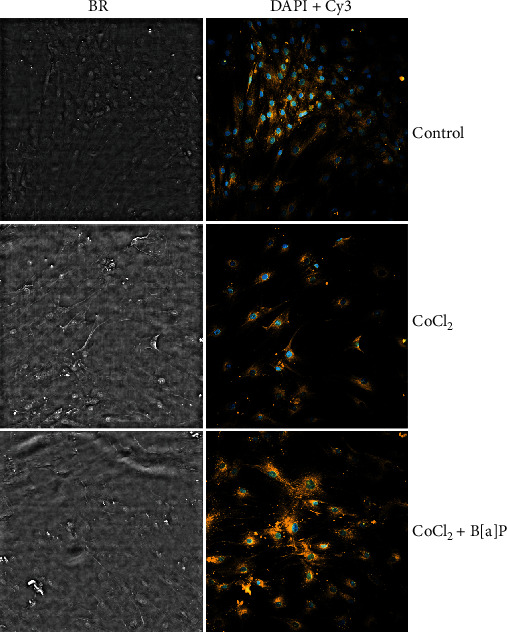
Images of primary cell culture of nonembolized meningioma (meningotheliomatous subtype). IN Cell Analyzer 2200, scale bar = 60 *μ*M, 200x magnification. The fluorescence images were obtained using Hoechst 33342 for *nuclei* (*blue*) and immunostained osteopontin (*yellow*), which is an oncogenic marker [36]. Abbreviations: CoCl_2_: 200 *μ*M CoCl_2_ in the incubation medium; BP+CoCl_2_: 200 *μ*M CoCl_2_+1 *μ*M B[a]P in the incubation medium; BF: bright-field mode; DAPI+Cy3: DAPI and Cy3 channels.

For qualitative characterization of the meningioma cell culture, oncogenic marker osteopontin was used.  Figure 4 shows cell culture images obtained via IN Cell Analyzer 2200 in bright-field mode and in DAPI and Cy3 channels. It is obvious that the live cells containing the oncogenic marker (osteopontin) are present both in the control cell culture and during incubation of the cells with the hypoxia activator CoCl_2_ and the AhR ligand B[a]P.

#### 3.2.2. Expression of *HIF-1α*, *AhR*, *ARNT*, and Target Genes of HIF-1*α* and AhR in Primary Cells from Patient-Derived Nonembolized Tumors

**Figure 5:**
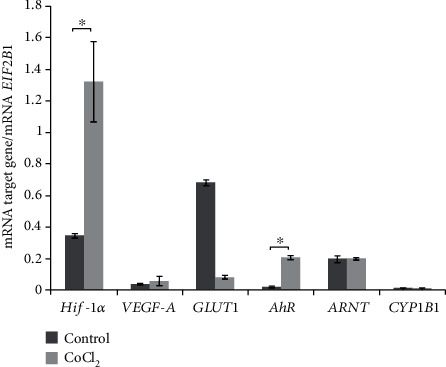
mRNA levels of *HIF-1α*, *AhR*, *ARNT*, *VEGF-A*, *GLUT1*, and *CYP1B1* in untreated meningioma cells and meningioma cells subjected to the “cobalt” model of hypoxia. The data are presented as the mean ± SE (*n* = 3); ^∗^*p* < 0.05. One-way analysis of variance (ANOVA) was performed, and the Bonferroni correction served as a post hoc test; *n* = 3 for all genes except *HIF-1α* in “cobalt” model samples (*n* = 4).

Figure 5 shows quantitative results on mRNA expression of genes *HIF-1α*, *VEGF-A*, *GLUT1*, *AhR*, *ARNT*, and *CYP1B1* in the cells that were exposed to 200 *μ*M CoCl_2_.The exposure of the patient-derived nonembolized tumor primary cells to hypoxic conditions—as expected—increased the expression of *HIF-1α* mRNA fourfold in comparison with the control cell culture but did not change the mRNA expression of HIF-1*α* target genes: *VEGF-A* and *GLUT1* (Figure 5). The decrease in the mRNA level of *GLUT1* was not statistically significant.

**Figure 6:**
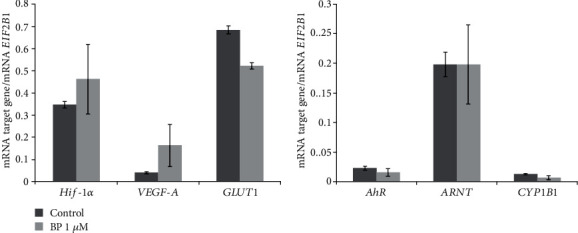
mRNA levels of HIF-1*α*, HIF-1*α* target genes (VEGF-A and GLUT1), AhR, ARNT, and an AhR target gene (CYP1B1) in nonembolized meningioma cells exposed to 1 *μ*M B[a]P. The data are presented as the mean ± SE (*n* = 3). One-way analysis of variance (ANOVA) was performed.

The exposure of the meningioma cell lines to chemically induced hypoxia increased the level of *AhR* mRNA ninefold as compared with the control cultured cells but did not alter the mRNA level of *ARNT* and of an AhR target gene (*CYP1B1*; Figure 5). To investigate a potential relation between the AhR and HIF-1*α* signaling pathways, we used AhR ligand B[a]P. When the cultured tumor cells were treated with 1 *μ*M B[a]P, there were no changes in mRNA expression of *HIF-1α*, its target genes, *ARNT*, *AhR*, and its target gene *CYP1B1* (Figure 6).

**Figure 7:**
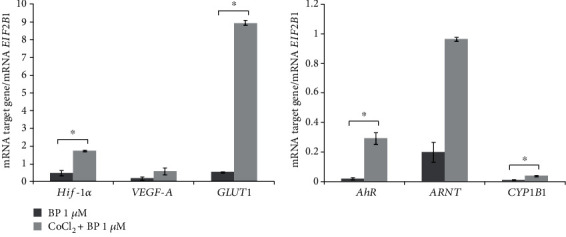
mRNA levels of *HIF-1α*, *VEGF-A*, *GLUT1*, *AhR*, *ARNT*, and *CYP1B1* in nonembolized meningioma cells exposed to CoCl_2_+B[a]P or to B[a]P. The mRNA levels of HIF-1*α*, AhR, ARNT, HIF-1*α* target genes (VEGF-A and GLUT1), and an AhR target gene (CYP1B1) in nonembolized meningioma primary cultured cells cotreated with 200 *μ*M CoCl_2_ and 1 *μ*M B[a]P or treated with 1 *μ*M B[a]P alone. The data are presented as the mean ± SE (*n* = 3). One-way analysis of variance (ANOVA) was performed, and the Bonferroni correction served as a post hoc test.

By contrast, the same concentration of B[a]P led to an increase in CoCl_2_-induced mRNA expression of both *AhR* and its target gene *CYP1B1* (Figure 7). In this case, the *AhR* mRNA level was 12 times higher than that in the cells without treatment with the hypoxia mimetic and B[a]P. During the cotreatment of the cells with CoCl_2_ and B[a]P, an increase in *ARNT* mRNA expression was noted too but was statistically insignificant. Cotreatment of the cells with CoCl_2_ and B[a]P also activated mRNA expression of *HIF-α* (fivefold) and *GLUT1* (Figure 7).

Thus, during the hypoxia simulated by means of CoCl_2_ in patient-derived tumor primary cultured cells, the expression of genes *HIF-1α* and *AhR* changed unidirectionally, whereas *AhR* and *CYP1B1* expressions were activated by the inducer (B[a]P) only during the hypoxic state of the cultured tumor cells.

## 4. Discussion

Hypoxia is one of the most common microenvironmental factors involved in carcinogenesis [39] and may play an important role in the development of aggressive characteristics of tumors [40]. Some studies have shown an association between the expression of *HIF-1α* (a key participant in the hypoxia signaling pathway) and meningiomas of different grades [41]. Relatively recently, *AhR* expression was detected in human brain meningiomas. It has been demonstrated that AhR signaling pathway components such as AhR and ARNT (also known as HIF-1*β*) were activated as meningioma malignancy progresses from low to high [42].

It is known that AhR is not only an environmental sensor but also a key regulator of physiological functions. The pleiotropic effects of AhR may, at least in part, arise from its ability to interact with other transcription factors [43, 44]. In particular, there is known crosstalk between AhR and HIF-1*α* in the case of their simultaneous activation in some human cell lines by various AhR ligands or hypoxia procedures. Nevertheless, these data are contradictory: while some studies have revealed mutual inhibition between HIF-1*α* and AhR pathways, others have shown that HIF-1*α* activation inhibits the AhR response but not vice versa [21].

To date, the potential crosstalk between AhR and hypoxia pathways in meningioma cells remains to be elucidated. For this reason and in an effort to better understand AhR signaling in meningioma, we analyzed benign intracranial meningiomas under hypoxic conditions such as preoperative endovascular embolization and under mimetic-induced hypoxia in short-term tumor cell culture to investigate the putative crosstalk between AhR and hypoxia pathways.

As revealed by our analysis of mRNA expression in samples of meningioma tissue subjected to hypoxic ischemic preconditioning, the expression of *HIF-1α* and its target genes (*VEGF-A*, *c-Myc*, and *GLUT1*) is not affected by this procedure, whereas *AhR*, *ARNT*, and *NcoA2* expressions significantly decreases.

We documented a heterogeneous pattern of expression of *HIF-1α* and its target genes in both nonembolized and embolized meningiomas; this finding may be due to the small sample size and different histological subtypes of the tumors under study. Perhaps, this is the reason why we did not see differences in *HIF-1α* expression between embolized and nonembolized tumors, although such differences have been reported previously [45]. On the other hand, as for mRNA expression levels of *AhR*, *ARNT*, and *NcoA2*, their decrease after embolization was statistically significant. Therefore, the hypoxia developing in benign meningioma as a consequence of endovascular embolization may interfere with the AhR signaling pathway at the level of *AhR* and *ARNT* mRNA expressions, and this effect does not depend on the histological structure of the meningioma.

Because the NcoA2 inhibits proteins of the bHLH/PAS family, such as HIF-1*α*, ARNT, and AhR [46], it is likely that in the molecular processes of meningioma ischemic hypoxia, AhR signaling probably plays a more important role than the HIF-1*α* pathway does. It is possible that the reduced level of *NcoA2* as a result of hypoxia suppresses the transactivation of the xenobiotic-sensitive element (XRE) of *AhR*, as demonstrated, for example, in HEK293T cells [24], and it is the nuclear receptor coactivator (NcoA2) that is the key trigger of molecular cascades in meningiomas subjected to ischemic hypoxia.

The inhibitory effect of meningioma embolization on the expression of AhR pathway genes and its regulator may have a long-term therapeutic effect on meningioma cells. In the absence of any exogenous ligand, AhR is necessary for proper cell proliferation [47]. As follows from the available literature data, AhR contributes to the progression of meningioma [47]. Consequently, any reduction in *AhR* expression in meningiomas will have a therapeutic effect. The decrease in *AhR* and *HIF-1α* mRNA levels in embolized meningiomas did not lead to a change in either HIF-1*α* or AhR protein amounts, thereby indicating a sufficiently high constitutive level of these proteins.

AhR regulates the cell cycle and apoptosis. Depending on the cell type, animal strain, experimental conditions, treatment regimen, timing of analysis, and duration of activation, AhR may cause either stimulation or inhibition of cell proliferation and of cell death [47]. The reasons for the heterogenicity of the AhR response are unclear at present. As follows from the available literature data, AhR contributes to the progression of meningioma [47]. Consequently, any decrease in *AhR* expression in meningioma cells, for example, as a consequence of embolization, can reduce the chances of tumor growth and progression. The decrease in *AhR* and *HIF-1α* mRNA levels in embolized meningiomas did not alter either HIF-1*α* or AhR protein amounts, thereby indicating a sufficiently high constitutive level of these proteins. CoCl_2_, a commonly used hypoxia-mimicking agent, artificially induces hypoxia and can block degradation of (and thus induce accumulation of) the HIF-1*α* protein [48, 49]. Many studies indicate that CoCl_2_ and hypoxia affect similar groups of genes in transcriptomic analyses [50].

According to our examination of mRNA expression in patient-derived meningioma primary cells, during the hypoxia induced by CoCl_2_, in addition to *HIF-1α* activation, there is AhR upregulation. mRNA expression of ARNT partner *HIF-1α*, *AhR*, AhR target (*CYP1B1*), and HIF-1*α* target genes did not change. mRNA expression of *HIF-1α*, *AhR*, *ARNT*, and *HIF-1α* and AhR target genes did not change when meningioma cells were incubated with B[a]P (AhR agonist). By contrast, in cells cotreated with B[a]P and CoCl_2_, the CoCl_2_-induced induction of *HIF-1α* and *AhR* mRNA expressions was found to be enhanced, and mRNA expression of *GLUT1* (HIF-1*α* target) and *CYP1B1* (AhR target) was activated.

According to the literature, hypoxia and hypoxia mimetics enhance the expression of ARNT in several human cancer cell lines, including prostate cancer (PC-3) cells, melanoma (518A2) cells, breast carcinoma (MCF-7) cells, hepatocellular carcinoma (Hep3B) cells, cervical adenocarcinoma (HeLa) cells, and multiple myeloma (H929) cells [51, 52]. In human breast cancer MCA-7 cells, hypoxia mimetic CoCl_2_ inhibits AhR-mediated processes [53]. In MCA-7 cells grown under CoCl_2_-induced hypoxia, 2,3,7,8-tetrachlorodibenzo-p-dioxin induction of CYP1A1 activity, a marker of AhR sensitivity, is significantly suppressed [53], and in human lung carcinoma A549 cells, HIF-1 induction weakens B[a]P-induced *CYP1A1* and *CYP1B1* mRNA expressions, and B[a]P attenuates the induction of a HIF-1 target: CA-IX [54]. The primary cultured cells from benign meningiomas in our study showed responses to the hypoxia mimetic (in terms of mRNA expression of *AhR*, *ARNT*, and an AhR target gene) that differ from those described in the literature; this discrepancy may be due to differences between the analyzed tissues.

Overall, our findings suggest that in intracranial benign meningioma, genes of the AhR signaling pathway are involved in the adaptation to hypoxia in both HIF-1*α*-dependent and HIF-1*α*-independent manners. It is possible that hypoxia in meningioma cells—depending on the conditions of its implementation and on the cellular microenvironment—can lead either to the depletion or to accumulation of key coregulatory proteins necessary for the execution of the AhR signaling pathway.

## Data Availability

Data is available upon request from the authors.

## Abbreviations

AhR: Aryl hydrocarbon receptor
ARNT: Aryl hydrocarbon receptor nuclear translocator
B[a]P: Benzo(*α*)pyrene
CoCl_2_: Cobalt chloride
HIF: Hypoxia-induced factor
HIF-1*α*: Hypoxia-induced transcription factor 1 subunit alpha
OPN: Osteopontin
PCR: Polymerase chain reaction
WHO: World Health Organization
WHOGr1: WHO grade 1
WHOGr2: Atypical grade.
